# The comparative effectiveness of Core versus Core+Enhanced implementation strategies in a randomized controlled trial to improve substance use treatment receipt among justice-involved youth

**DOI:** 10.1186/s12913-022-08902-6

**Published:** 2022-12-16

**Authors:** Danica K. Knight, Steven Belenko, Michael L. Dennis, Gail A. Wasserman, George W. Joe, Gregory A. Aarons, John P. Bartkowski, Jennifer E. Becan, Katherine S. Elkington, Aaron Hogue, Larkin S. McReynolds, Angela A. Robertson, Yang Yang, Tisha R. A. Wiley

**Affiliations:** 1 Karyn Purvis Institute of Child Development, Texas Christian University, Fort Worth, USA; 2 Department of Criminal Justice, Temple University, Philadelphia, USA; 3 Lighthouse Institute, Chestnut Health System, Chicago, USA; 4 Department of Psychiatry, College of Physicians and Surgeons, Columbia University, New York, USA; 5 Institute of Behavioral Research, Texas Christian University, Fort Worth, USA; 6 Child and Adolescent Services Research Center, University of California, San Diego, USA; 7 Department of Sociology, University of Texas at San Antonio, San Antonio, USA; 8 Partnership to End Addiction, New York, USA; 9 Mailman School of Public Health, Columbia University, NYS Psychiatric Institute, New York, USA; 10 Social Science Research Center, Mississippi State University, Starkville, USA; 11 Service Research Branch, National Institute on Drug Abuse, Bethesda, USA

**Keywords:** Juvenile justice, Services cascade, Implementation intervention, Substance use treatment, EPIS, Interorganizational collaboration, System change

## Abstract

**Background:**

Most justice-involved youth are supervised in community settings, where assessment and linkage to substance use (SU) treatment services are inconsistent and fragmented. Only 1/3 of youth with an identified SU need receive a treatment referral and even fewer initiate services. Thus, improving identification and linkage to treatment requires coordination across juvenile justice (JJ) and behavioral health (BH) agencies. The current study examines the comparative effectiveness of two bundled implementation intervention strategies for improving SU treatment initiation, engagement, and continuing care among justice-involved youth supervised in community settings. Exploration, Preparation, Implementation, Sustainment (EPIS) served as the conceptual framework for study design and selection/timing of implementation intervention components, and the BH Services Cascade served as the conceptual and measurement framework for identifying and addressing gaps in service receipt.

**Methods:**

Part of a larger Juvenile-Justice Translational Research on Interventions for Adolescents in the Legal System (JJ-TRIALS) Cooperative, this study involved a multisite, cluster-randomized control trial where sites were paired then randomly assigned to receive Core (training teams on the BH Services Cascade and data-driven decision making; supporting goal selection) or Core+Enhanced (external facilitation of implementation teams) intervention components. Youth service records were collected from 20 JJ community supervision agencies (in five states) across five study phases (baseline, pre-randomization, early experiment, late experiment, maintenance). Implementation teams comprised of JJ and BH staff collaboratively identified goals along the BH Cascade and used data-driven decision-making to implement change.

**Results:**

Results suggest that Core intervention components were effective at increasing service receipt over time relative to baseline, but differences between Core and Core+Enhanced conditions were non-significant. Time to service initiation was shorter among Core+Enhanced sites, and deeper Cascade penetration occurred when external facilitation (of implementation teams) was provided. Wide variation existed in the degree and nature of change across service systems.

**Conclusions:**

Findings demonstrate the criticality of early EPIS phases, demonstrating that strategies provided during the formative exploration and preparation phases produced some improvement in service receipt, whereas implementation-focused activities produced incremental improvement in moving youth farther along the Cascade.

## Contributions to the literature

- Compares the effectiveness of two bundles of implementation strategies for improving substance use treatment initiation, engagement, and continuing care among justice-involved youth when cross-system collaboration is required;
- Combines two conceptual frameworks to guide study components and design: EPIS (selection and timing of intervention and data collection components) and Behavioral Health Services Cascade (focus of goals selection and data-driven decision-making activities).
- Examines change across study phases, enabling comparisons with baseline service receipt rates and potential sustainment of gains;
- Includes data from 20 service systems in five US states, involving both juvenile justice and behavioral health agencies.

## Introduction

A high proportion of youth involved in juvenile justice (JJ) exhibit problematic levels of alcohol and other substance use (SU). For this reason, and because SU services are linked to outcomes including reduced recidivism [1, 2], addressing SU has become a focus of JJ agency efforts to lower recidivism and address youth needs. While some agencies provide SU treatment directly, most refer youth to external service providers [3], which requires cross-system collaboration in identifying and addressing SU needs [4, 5]. The current study, from the Juvenile Justice-Translational Research on Interventions for Adolescents in the Legal System (JJ-TRIALS) Cooperative, examines the effectiveness of two implementation intervention bundles for promoting SU service uptake within complex systems involving JJ and behavioral health (BH) agencies.

### The need for system change

Substance use is common among JJ-involved youth and is a primary factor associated with re-offending [2]. Approximately 51% have substance problems requiring treatment, including 49% for marijuana, 25% for alcohol, and 18% for other drugs [3, 6, 7]. Identification of a SU treatment need typically occurs as part of JJ intake and assessment procedures and a referral for treatment follows [8]. Most adjudicated youth are supervised and referred to services in community settings [3]. This intersection of justice and health agencies is where appropriate clinical assessment and linkages to treatment services are inconsistent and fragmented at best [9]. Youth with serious SU issues are not likely to access treatment [10] and have relatively low retention after initiation, especially compared with adults [11, 12]. Data from US samples of JJ-involved youth indicate that among youth in need of SU services, 31% receive a treatment referral, 21% initiate treatment, 10% engage for at least 6 weeks, and 6% continue in care (at least 90 days); receiving a referral significantly increases the likelihood of initiation [13].

Cascade frameworks depict a continuum where clients move through a series of service steps to maximize clinical gains [14–16]. The further an individual “penetrates,” the greater the likelihood of service receipt and improvement in BH outcomes. The BH Services Cascade (hereafter Cascade [14]) serves as a conceptual and measurement framework for identifying gaps in service receipt for youth under community supervision and includes six phases: screening/assessment, identification of need, referral, initiation, engagement, and continuing care. Completion of earlier steps is generally required to complete subsequent steps; a referral is unlikely if the agency has not identified a need for treatment [13]. The Cascade framework is useful for guiding organizations toward identifying youth with SU problems and linking youth to clinical services, defining data needed to understand where in the service continuum gaps exist, and tracking the impacts of interventions to reduce unmet service needs [14].

### Implementing change across complex Systems of Care

A prominent model for promoting system change, Exploration, Preparation, Implementation, Sustainment (EPIS) [17, 18], serves as the conceptual framework for the JJ-TRIALS implementation intervention and study design [19]. In addition to clearly delineated phases, EPIS recognizes the multi-level nature of service delivery in which multiple actors/agents are nested and function synergistically within and between organizations, systems, and the broader environmental contexts. Further, EPIS is intentionally flexible to address implementation across diverse systems and contexts in both linear and cyclical (recursive) processes [20]. In the current study, site-specific data were used during Exploration to identify service gaps along the Cascade, and interorganizational teams (representatives from JJ and BH) identified goals and potential new practices. During Implementation, sites applied data-driven feedback loops independently (Core) or with external support (Core+Enhanced) to monitor and evaluate progress toward goals. While numerous implementation intervention strategies show promise for promoting system change [21], JJ-TRIALS focused on select components: interorganizational collaboration, data-driven decision making (including needs assessment, site feedback reports, and support for goal selection), and external facilitation of local implementation teams.

Prior research confirms the value and effectiveness of interorganizational relationships among agencies serving JJ-involved individuals [4, 22, 23]. Where JJ-involved youth are concerned, collaborating agencies include youth-oriented mental health providers, drug treatment agencies, juvenile courts, etc. [8, 24–26], and effective interorganizational collaboration requires information exchange, cross-agency client referrals, networking protocols, interagency councils, and integrated services [27]. Collaborations flourish when targeted goals and specific roles for each agency are clearly defined and bidirectional communication occurs [4]. Improvement efforts are most effective when interorganizational teams are formed [28, 29], teams include stakeholders with diverse expertise representing internal and external interests, and local champions are identified and equipped to implement and sustain change [30].

Data-driven decision making (DDDM) involves using data, collected within the specific context and in collaboration with stakeholders (e.g., agency staff, clients), to systematically evaluate the effectiveness of change efforts and to inform practice and policy within organizations and systems. DDDM has been adopted by different fields that include education, healthcare, and criminal justice [31–34] and can include quantitative (e.g., agency service records) and qualitative (e.g., staff perceptions of feasibility) data. A common form, Plan-Do-Study-Act (PDSA) [35], includes an iterative process involving small-scale testing and is most effective when teams utilize data that are highly relevant to the target outcomes. While most JJ systems collect administrative records on “front-end” Cascade activities (e.g., screening, referral), the collection of “back-end” treatment-specific records (e.g., initiation, engagement) is inconsistent or non-existent. This is especially so for services that are delivered by community providers which often involve a “handoff” from JJ to BH [3, 13].

External facilitation of local implementation teams can be beneficial in supporting change, encouraging the adaptation of best practices in a manner that suits the particular qualities of the organization or system [36, 37]. Facilitation is particularly helpful when implementation teams have limited expertise in using data to inform practice; goals include transforming interorganizational business practices; or collaborating agencies have not yet established effective communication, shared language, and joint priorities. The practice of evaluating change efforts in real time is often best introduced by a knowledgeable facilitator and is linked to positive subjective and objective performance outcomes [38]. While robust effects of facilitation may be observed when long-term implementation oversight is provided, training local champions in leadership is also critical if new practices are to be sustained.

System change occurs within a broader context, yet information regarding inner and outer context factors that potentially predict treatment receipt among JJ youth is limited. An examination of Cascade outcomes during the baseline period of JJ-TRIALS (before the intervention began) found that elevated job stress, greater intra-agency communication, and more collaborative practices with community providers were associated with lower initiation rates, perhaps due to increased agency efforts to address problems [5]. Such factors may be important drivers of treatment initiation and perhaps other downstream Cascade outcomes as implementation teams work to address issues over time.

### The current study

The current study examined the comparative effectiveness of two bundled implementation intervention strategies for improving receipt of SU services along the BH Services Cascade. Prior reports using JJ-TRIALS data described Cascade outcomes covering the baseline period before intervention began [5, 13] and the intervention’s impact on referral to BH services [39]. Here, the impact on later Cascade activities (service initiation, treatment engagement, and continuing care) was examined using a subset of sites with data across the full Cascade (*N* = 20). It was expected that participation in a Core set of strategies would result in improvement over time and that sites receiving Core+Enhanced strategies (primarily external facilitation in the application of DDDM) would show greater improvement compared to sites in the Core-only condition. Change over time in service receipt was measured at the agency/community level through nested youth cohorts that correspond with five study periods and align with EPIS: Baseline, Pre-Randomization, Early Experiment, Late Experiment, Maintenance. The following Hypotheses were tested:

- H1: The percentage of youth receiving target services (Initiation, Engagement, Continuing Care) will increase in both Core and Core+Enhanced conditions between Baseline and Late Experiment study phases.
- H2: Compared to Core sites, Core+Enhanced sites will have greater increases in the percentage of youth receiving target services between Baseline and Late Experiment study phases.
- H3: Among youth with a treatment referral, youth in Core+Enhanced sites will initiate services more quickly compared to youth in Core sites over time (i.e., time to initiation will be shorter during Late Experiment compared to Baseline).
- H4: Youth in both Core and Core+Enhanced sites will progress further in the Behavioral Health Services Cascade over time (i.e., Cascade Penetration means will be higher during Late Experiment compared to Baseline).
- H5: Youth in Core+Enhanced sites will progress further in the Behavioral Health Services Cascade over time compared to youth in Core sites (i.e., penetration means will be higher during Late Experiment compared to Baseline).

In addition to H1-H5, it was expected that means on Cascade outcomes would remain constant between Late Experiment and Maintenance study phases, after expert facilitation ceased (change driven by the research design).

## Method

### Research design

Data were from the JJ-TRIALS cooperative agreement, funded by the National Institute on Drug Abuse. JJ-TRIALS included six research centers, each working with six local justice agencies providing community justice supervision (e.g., probation) in Florida, Georgia, Kentucky, Mississippi, New York, Pennsylvania, and Texas [19]. Research centers received study approval from their institutional review boards, and a waiver of consent was granted to review deidentified youth records. Thirty-six sites were recruited for participation in the parent randomized control trial. Within states, sites were matched into pairs based on JJ agency characteristics (e.g. number of juvenile referrals, number of probation officers, county population) and each pair was randomly assigned to one of three start dates for logistical purposes. All sites received Core intervention strategies, and one site within each pair was randomly assigned to receive Core+Enhanced strategies [19]. Due to limitations in existing youth records data [13], the current study is limited to data from the 20 sites that captured services along the entire Cascade through continued care. Compared to youth in sites without treatment initiation data [39], youth in this sample were more likely to be non-white, on probation, positive on urinalysis, higher charge level (felony), and to be in urban settings with lower poverty.

### Participants

Youth records data were drawn from 20 JJ agencies (10 matched pairs) across five states [13]. Because not all youth exhibit SU problems (and therefore are not expected to move through the Cascade), data were restricted to youth with an identified SU need (e.g., positive SU screen, clinical assessment). Data were limited to the youth’s first referral to JJ in addressing participant outcomes in this study. The resulting sample included 8988 youth (*N* = 141 to 2017 per site; Core *n* = 56 to 1210; Core+Enhanced *n* = 59 to 970). Overall totals by intervention for these treatment sites randomly assigned in pairs were Core (*n* = 4587, 51%) and Core+Enhanced (*n* = 4401, 49%). Youth cohort assignment was based on date of entry into the JJ system. With regard to Study Phase, 3242 (36.1% of the sample) started in Baseline, 1894 (21.1%) started in Pre-randomization, 1399 (15.6%) started in Early Experiment, 1314 (14.6%) started in Late Experiment, and 1139 (12.7%) started in Maintenance (mutually exclusive cohorts).

### Procedures 

The study protocol has been previously published [19]. All sites received Core implementation intervention activities which included the creation of implementation teams involving representatives from JJ and at least one BH agency, training on the Cascade (provided to agency leadership, team members, and staff with direct youth contact) [5], support for goal selection (based on site-specific feedback reports) [40], and training on applying DDDM [41]. Implementation teams in Core sites were encouraged to apply these strategies on their own.

The Core+Enhanced arm included all components delivered as part of the Core intervention plus active facilitation of process improvement efforts [19]. During Implementation (Early/Late Experiment periods), external facilitators guided agency efforts to develop plans, monitor progress, and sustain changes using rapid-cycle testing; assisted implementation teams in the design and production of periodic reports (using JJ data) in accordance with DDDM training; coached leaders in the management of team activities and roles; attended meetings throughout the Preparation and Implementation phases; and transitioned leadership responsibilities to implementation team members over time. To ensure fidelity, intervention and protocol compliance were monitored by the JJ-TRIALS Coordinating Center, with feedback provided at regular intervals [19].

### Measures 

Site Pairs referred to matched pairs of sites. Study Phase referred to one of five study periods corresponding to EPIS: 1 = Baseline (Exploration), 2 = Pre-randomization (Preparation), 3 = Early Experiment (Implementation), 4 = Late Experiment (Implementation), and 5 = Maintenance (Sustainment) [20]. All phases were 6 months in duration, with the exception of Baseline (6–12 months). The Experiment period was divided into two because early months were typically spent improving data quality and developing, adapting, and/or piloting procedures prior to scaling up. Each Study Phase consisted of independent samples of youth classified into cohorts based on their date of referral to the JJ agency [19].

Youth demographic and offense characteristics were extracted from justice case management records and included gender, race (categories listed in Table 1 were dichotomized as 1 = White or 0 = non-White), Hispanic ethnicity, age, positive urine screen (1 = yes, 0 = no), probation status, alcohol or drug related charges, and maximum charge level (1 = felony, 2 = misdemeanor, 3 = summary/citation, or 4 = status offense). For Community Supervision: 2 = any probation, parole, Juvenile Drug Court, detention, or other justice status; 1 = other community supervision or diversion; 0 = other combinations or no status indicated. Higher supervision level included ongoing formal oversight by probation, parole, or juvenile drug court authorities; lower-level reflected diversion to community programs or placement on informal community supervision [5, 42]. Change in supervision status or intensity was not captured. County characteristics included Urbanicity (percentage of residents in urban areas) and Poverty (percentage of families with youth living in poverty).

**Table 1:** Sample characteristics

	Total Sample		Core		Core + Enhanced		ω^2^ for total sample ^c^
Characteristics	N	Mean (SE)	N	Mean (SE)	N	Mean (SE)	
**Youth characteristics**							
Age	8987	15.25 (1.33)	4586	15.27 (1.33)	4401	15.24 (1.34)	.00 (.000, .001)
Male (%)	8985	79%	4586	81.36%	4399	77.20%	.003 (.001, .005)
White	8567	52%	4190	41.03%	4377	62.1%	.044 (.036, .053)
Non-White	8567	48%	4190	59.0%	4377	37.9%	.044 (.036, .053)
Black (%)	8567	46%	4190	56.78%	4377	35.64%	.045 (.037, .054)
Hispanic (%)	7920	30%	3709	20.90%	4211	37.97%	.035 (.027, .043)
Percentage of youth had a positive urine screen (Positive Urine)	8988	28%	4587	22%	4401	34%	.017 (.012, .023)
Charges ^a^	8508	1.60 (.55)	4356	1.58 (.55)	4152	1.62 0.55)	.001 (.0003, .003)
Maximum Charge Level	8409	1.60 (.55)	4313	1.58 (.55)	4096	1.63 (.55)	.0015 (.0003, .0037)
Current charges were alcohol or other drug-related (%)	8988	34%	4587	36%	4401	32%	.001 (.0002, .003)
Current charges were violence-related (%)	8988	22%	4587	19%	4401	24%	.003 (.002, .006)
Level of community supervision ^b^	8988	1.63 (.52)	4587	1.60 (.55)	4401	1.67 (.49)	.004 (.002, .008)
Juvenile justice status (probation, %)	8988	65%	4587	63%	4401	67%	.002 (.001, .004)
**County characteristics**							
Percentage of residents in urban areas (% Urbanicity)	8988	92%	4587	92%	4401	92%	.002 (.0004, .004)
Percentage of families with youth living in poverty	8988	18%	4587	19%	4401	17%	.033 (.026, .040)

*Note*. ^a^ Charges = the total number of charges in the following categories: alcohol and drug-related charges, probation/parole violation, weapons offense, and other status offense [13]. ^b^ Level of community supervision includes 2 = more community supervision (any probation, parole, Juvenile Drug Court, detention, or other justice status), 1 = less (other community supervision or diversion), and 0 = other combinations of statuses or no status indicated. ^c^ omega^2^ (ω^2^) was used to evaluate significance and select covariates for subsequent analyses, with the following designations: no effect (ω^2^ = 0), small effect (ω^2^ = .01), medium effect (ω^2^ = .06), and large effect (ω^2^ = .14)

Cascade measures were constructed in accordance with prior studies [5, 13, 19, 39]: (a) referred to JJ, (b) received screening/assessment, (c) identified SU need, (d) referred to SU treatment, (e) initiated SU treatment, (f) engaged in SU treatment, and (g) received continuing care SU services. For each youth, receipt of each service was scored as a dichotomy (1 = yes, 0 = no). Referred to JJ included the total number of referrals to the JJ system for the time period (i.e., cohort). Screened was defined as having been administered a SU screening instrument. Identified SU Need was based on results from screening, urinalysis, clinical assessment, or other sources of information (e.g., self-disclosure); a positive (or above threshold) score on one or more was coded as yes. Referral to Treatment included any referral for SU services (e.g., inpatient, residential, outpatient, individual counseling). Initiation was coded yes if treatment start date or attendance at one or more sessions existed in the record. Engagement was defined as staying in treatment for at least 6 weeks (discharge date minus start date). Continuing Care was defined as more than 90 days of services (e.g., continuation of initial service or step down to aftercare). Treatment Services was the sum of Initiation, Engagement, and Continuing Care. To minimize missing data, each service was coded as “yes” or “other” (no, legitimate skips, missing data) [13].

Time to Service was determined by the number of days between receipt of services (e.g., referral to and initiation of SU treatment). Cascade Penetration represented the last Cascade stage that an individual achieved in their first referral to the JJ system: 0 = no Cascade stage, 1 = Screened/assessed, 2 = Identified Need, 3 = Referred, 4 = Initiated, 5 = Engaged, and 6 = Continued in Care.

#### Analytic plan

Because youth were nested within site and sites were randomly assigned to condition within each matched pair, a multilevel matched block analysis was used to test hypotheses using SAS PROC MIXED [43], with Site Pairs used as a blocking factor for H4 and H5. For H1 and H2, SAS PROC GLIMMIX [43] was used because the dependent variables were dichotomies. For H1 and H4, models included only Study Phase (differences in cohorts over time). For H2 and H5, models involved Study Phase, Condition (Core vs. Core+Enhanced), and their interaction. Effect sizes for mean differences [44] and overall effect sizes for models [45] were calculated [46]. Effect sizes were calculated for the multilevel analyses using Lorah’s (2018) approach (*f* ^*2*^*)*, where .02 is a small, .15 a medium, and .35 a large effect size, respectively.

Covariates (see Table 1) were selected using a step-wise multiple regression analysis with Treatment Services as the dependent variable. Because the sample was large, effect size (partial omega^2^; *ω*^2^) was used to evaluate significance, with the following designations: no effect (*ω*^2^ = 0), small effect (*ω*^2^ = 0.01), medium effect (*ω*^2^ = 0.06), and large effect (*ω*^2^ = 0.14). The partial *ω*^2^ showed that Race/ethnicity and Poverty had values greater than 0.03 (between small and medium). JJ Status and Maximum Charge Level showed a small effect size (at least 0.02). These covariates were used to control for case mix differences between the matched site pairs.

Survival analysis was used to examine time to initiation by Condition and Study Phase (H3). Cox proportional hazard model (SAS PROC PHREG) was chosen because it allows censored observations (treatment initiation status was unknown for some youth when the study ended), assumes a parametric form for the predictors, and allows for an unspecified form for the underlying survivor function. For interpretation of the estimated maximum likelihood (ML) weights, corresponding hazard ratios were computed by exponentiating the ML weights. While the hazard is defined as the slope of the survival curve (i.e., how rapidly participants reach the event), the hazard ratio compares the hazards for two categories (groups), that is, the relative survival experience of the two groups. For example, if the hazard ratio is 2.0, then the rate of achieving the event in one categorical group is twice the rate of achieving the event in the other group.

## Results

### Sample description

As noted above, characteristics that were significantly correlated with the treatment services were considered as potential covariates in the analyses. Among the total sample of youth with a need for SU treatment (*N* = 8988), 7817 (87%) received Screening, 2252 (25.1%) received a treatment Referral, 1763 (19.6%) Initiated, 681 (7.6%) Engaged, and 359 (4.0%) Continued in Care. With regard to average conditional probabilities, 78.3% of Referred youth Initiated, 38.6% of Initiated youth Engaged, and 52.7% of Engaged youth Continued in Care. On average, 16 days elapsed between JJ Intake and Screening (*n* = 7766 of 8988; *M *= 15.74, *SD* = 38.57), 130 days between Screening and Referral (*n* = 1687 of 8988; *M* = 129.61, *SD* = 125.02), and 156 days between Screening and Initiation (*n* = 1670 of 8988; *M* = 155.59, *SD* = 131.29). Percentages of youth receiving services were comparable to those reported in other studies of the full sample at baseline under the same Cooperative [5, 13]. Regarding Cascade penetration, of 1763 who Initiated treatment, 1083 (61.4%) went no further in the Cascade, 322 (18.3%) achieved Engagement, and 358 (20.3%) achieved Continuing Care in successive steps.

### Initial base model

The base models for each dependent variable revealed significant estimates for site variances for the three treatment services. Nineteen percent of Initiation, 11% of Engagement, and 7% of Continuing Care were due to the variance between sites for the paired agencies, confirming the need for multilevel analyses.

#### H1: service receipt over time, across conditions

H1 examined whether the percentage of youth who Initiated, Engaged, and entered Continuing Care differed between Study Phases (see Table 2). For Initiated, Baseline versus Early Experiment (difference = − 0.0568, *t* = − 4.82, *p <* 0.0001) represented a 5.68% increase and Baseline versus Late Experiment (difference = − 0.0582, *t* = − 4.80, *p <* 0.0001) represented a 5.82% increase (see Table 3). When Early and Late Experiment (*M* = .115, *SE* = .019) were combined, the contrast with Baseline was also significant (*M*  = .057, *SE*  = .010; *t* = 5.92, *p <* 0.0001), implying a total 5.7% increase between the Baseline and Early/Late Experiment cohorts.

**Table 2:** H1: Type III Fixed Effects for Treatment Services while Controlling for Covariates

Source	Treatment Initiation^**a**^		Treatment Engagement^**b**^		Continuing Care^**c**^	
	*df*	*F-test (p)*	*df*	*F-test (p)*	*df*	*F-test (p)*
Site pairs	9,10	0.53 (.82)	8,8	**4.09 (.03)**	8,7	.97 (.52)
Study Phase	4,7973	**10.29 (< .0001)**	4,6010	**10.55 (< .0001)**	4,5924	**9.53 (< .0001)**
Male	1,7973	3.06 (.09)	1,6010	0.03 (.87)	1,5924	0.04 (.85)
White	1,7973	**17.21 (< .0001)**	1,6010	**6.07 (.01)**	1,5924	**5.58 (.02)**
Probation	1,7973	**200.50 (< .0001)**	1,6010	**76.82 (< .0001)**	1,5924	**58.40 (< .0001)**
Positive Urine	1,7973	**41.80 (< .0001)**	1,6010	**24.87(< .0001)**	1,5924	**19.31 (< .0001)**
Maximum Charge Level	1,7973	0.15 (.71)	1,6010	1.41 (.24)	1,5924	2.10 (.15)
% Urbanicity	1,7973	0.67 (.42)	1,6010	**5.16 (.03)**	1,5924	**4.37 (.04)**

*Note.* Probation = Whether or not being on probation. Positive Urine = Whether or not having a positive urine screen. % Urbanicity = Percentage of residents in urban areas. Bold means *p* < .05

^a^ Treatment Initiation: *χ*^*2*^(1) = 1738.63, *p* < 0.0001, site variance: 0.03 (0.01), *Z* = 3.00, *p* = 0.001, residual variance: 0.13 (0.002), *Z* = 66.96, *p* < 0.0001, ES = .01

^b^ Treatment Engagement: *χ*^*2*^(1) = 551.77, *p* < 0.0001, site variance: 0.01 (0.004), *Z* = 2.72, *p* = 0.003, residual variance: 0.08 (0.001), *Z* = 59.17, *p* < 0.0001, ES = .01

^c^ Continuing Care: *χ*^*2*^(1) = 264.71, *p* < 0.0001, site variance: 0.003 (0.001), *Z* = 2.47, *p* = 0.007, residual variance: 0.05 (0.001), *Z* = 58.60, *p* < 0.0001, ES = .03

**Table 3:** Contrasts among Study Phases for Treatment Services (controlling for covariates; H1)^a^

Study Phase (*M*, *SE*) ^b^	***t***	***p***	Lower 95% CI	Upper 95%CI	Effect size ***d***
*Reference*: Baseline (0.15, 0.07)	**Treatment Initiation**				
Early Experiment (0.20, 0.07)	−4.82	**<.0001**	0.03	0.08	**0.06**
Late Experiment (0.21, 0.07)	−4.80	**<.0001**	0.03	0.08	**0.06**
Maintenance (0.15, 0.07)	−0.34	0.74	−0.02	0.032	0.00
*Reference*: Baseline (0.04, 0.03)	**Treatment Engagement**				
Early Experiment (0.07, 0.03)	−2.38	**0.02**	0.01	0.05	**0.03**
Late Experiment (0.04, 0.03)	−0.10	0.927	− 0.02	0.02	0.00
Maintenance (−0.01, 0.03)	4.37	**< 0.0001**	−0.07	−0.03	**0.05**
*Reference*: Baseline (0.01, 0.03)	**Continuing Care**				
Early Experiment (0.03, 0.03)	1.64	0.10	−0.001	0.03	**0.01**
Late Experiment (0.01, 0.03)	0.65	0.52	−0.02	0.01	**0.01**
Maintenance (−0.03, 0.03)	4.68	< **0.0001**	−0.06	−0.02	**0.04**

*Note*. ^a^ *χ*^*2*^(1) = 66.95, *p* < 0.0001, site variance = 0.08 (0.04), *Z* = 2.17, *p* = 0.02; residual variance = 0.44 (.01), *Z* = 66.95, *p* < .0001

^b^ Least Square Means (Standard Errors). 95% CI = 95% confidence intervals for the differences between least square means for the contrasts. The contrast between Baseline Phase and Pre-Randomization Phase was omitted because it was not involved in hypothesis testing. Treatment Initiation ES = .013; Treatment Engagement ES = .033; Continuing Care ES = .032. Effect size *f*^*2*^ is an unbiased estimate of how much variance in the characteristic is accounted for by the condition (Core vs Core+Enhanced) in predicting the dependent variable

For Engagement, differences were significant between Baseline and Early Experiment (difference = 0.0268, *t* = − 2.38, *p =* 0.01) but not Late Experiment (difference = 0.00119, *t* = − 0.10, *p =* 0.92). When Early and Late Experiment were combined, contrasts were not significant (*M* = .014, *SE* = .01; *t* = 1.54, *p* = .13), representing only an increase of 1.4%. While Baseline versus Maintenance was significant (*t* = 4.37, *p <* 0.0001), it represented a decrease.

For Continuing Care, Baseline to Early Experiment (*t* = − 1.64, *p* = .10) and Late Experiment (*t* = 0.65, *p* = .52) were not significant. When Early and Late Experiment were combined, the contrast with Baseline was not significant (*M* = .009 = .004, *SE* = .014 = .007; *t* = .63, *p* = .53). Conversely, Baseline versus Maintenance was significant (*M* = −.03, *SE* = .03; *t* = 4.68, *p* <  0.0001), representing a significant decrease.

In summary, there was some statistical support for H1. The cohort corresponding to Early Experiment had significantly higher Initiation and Engagement compared to Baseline, but not Continuing Care. However, increases from Baseline did not extend to Maintenance, and results for the Late Experiment group were mixed.

#### H2: service receipt over time, by condition

H2 examined differences between Conditions over time, with Site Pairs as the blocking factor. The estimates of the site variation for each treatment service variable (Initiated: 0.03 (0.02), *Z* = 2.08, *p* = 0.02; Engaged: 0.01 (0.004), *Z* = 1.75, *p* = 0.04; Continuing Care: 0.004 (0.003), *Z* = 1.60, *p* = 0.05) were significant; residual variances were also significant (Initiated: 0.13 (0.002), *Z* = 66.93, *p* <  0.0001; Engaged: 0.08 (0.001), *Z* = 59.14, *p* <  0.0001; Continuing Care: 0.05 (0.001), *Z* = 58.57, *p* <  0.0001). The Type III Fixed Effects revealed that Study Phase was significant for each outcome [Initiated F(4, 8067) = 9.75, *p <* 0.0001; Engaged F(4, 6104) = 9.87, *p <* 0.0001; Continuing Care F(4, 6009) = 9.36, *p <* 0.0001], but neither the main effect for Condition nor its interaction with Study Phase was significant. Therefore, there was no statistical support for Hypothesis 2; the increase between Early Experiment and Baseline did not differ significantly by condition.

**Fig. 1:**
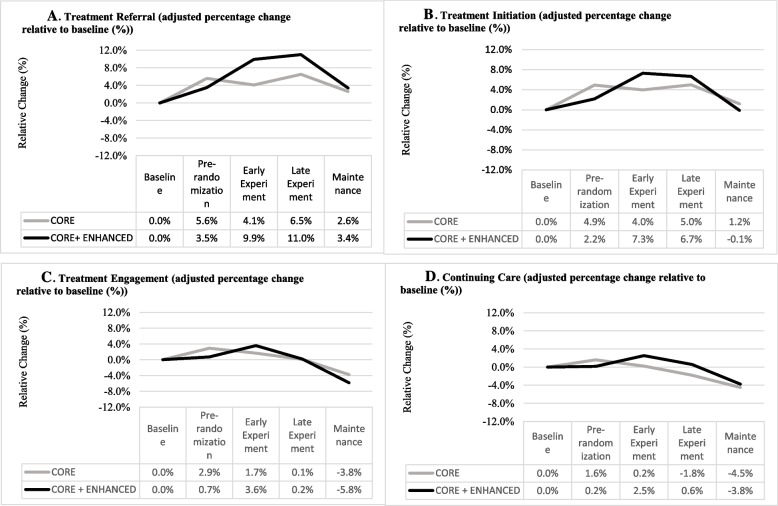
Relative change of adjusted percentage to baseline for each Cascade stage across Implementation phases *Note.* The interaction between Condition and Study Phase was not significant in any of the four dependent variables depicted in these four panels

Change across Study Phases relative to baseline is depicted in Fig. 1. Findings reported in Panel A (Treatment Referral) replicate those reported with the full JJ-TRIALS sample [39]. The pattern in Panel B (Initiation) is similar to referral, with non-significant differences between Core and Core+Enhanced conditions. The “decay” seen during Maintenance was consistent across outcomes and may be attributed (in part) to incomplete data in latter cohorts (truncated time frame due to end of the study).

#### H3: time to initiation

For H3, a Cox proportional hazard model was used to analyze time to treatment initiation on Site pair blocks, Condition, Study Phase, Condition x Study Phase, and covariates. Of the 2145 youth who initiated, 1773 had complete data on covariates; 1282 had an event (time to initiation); and 491 were right censored, with no initiation by the end of the data collection.

**Fig. 2:**
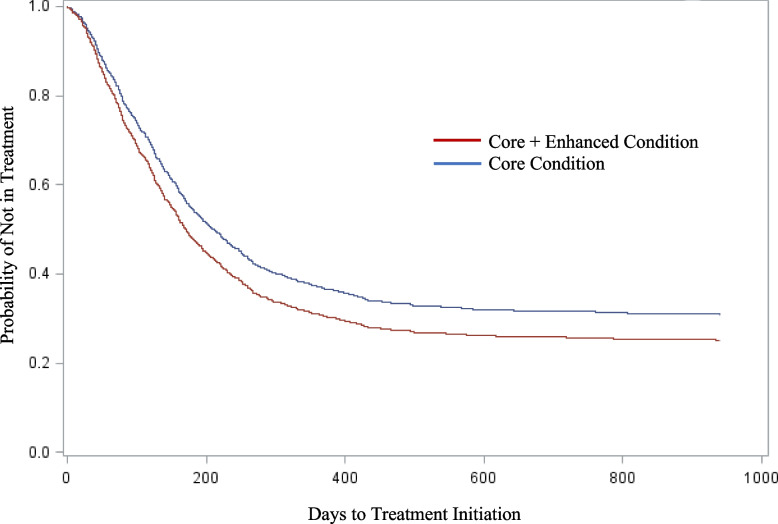
Survival curves for days to treatment initiation by condition

For this model, the Joint Tests showed Site Pairs [*χ* ^2^(9) = 538.59, *p* < 0.0001] and Condition [*χ*^2^(1) = 5.59, *p =* 0.0180] were significant, indicating that time to initiation (survival curves) was significantly different for Core and Core+Enhanced (Fig. 2). However, neither Study Phase [*χ* ^2^ (4) = 3.21, *p* = 0.52] nor Condition x Study Phase [*χ* ^2^(4) = 2.04, *p* = 0.73] were significant. Of the covariates, only positive urinalysis was significant [*χ* ^2^(1) = 17.22, *p* < 0.0001].

The maximum likelihood estimated weight for Core, [*b* = − 0.41 (.18), *χ* ^2^(1) = 5.59, *p* = .018], corresponded to a hazard ratio (HR) of 0.66. Therefore, there is a 33.9% reduction in achieving treatment initiation for Core compared to Core+Enhanced. The maximum likelihood estimated weight for Core+Enhanced was b = 0.41 (0.18), with a HR = 1.51. For Core+Enhanced the rate of achieving treatment initiation is approximately 1.51 times that for Core; that is, a 51.3% increase in achieving initiation. The HR for Core would suggest that the hazard (or shape of the survival curve) is two-thirds of that for Core+Enhanced. In summary, youth in Core+Enhanced initiated treatment more quickly than those in Core (Fig. 2).

### Initial base model for penetration

H4 and H5 assessed the maximum Penetration along the Cascade. Results of the base model found treatment site variance to be significant [*χ* ^2^ (1) = 1529.96, *p* < 0.0001, with site variance of 0.18(0.06), *Z* = 3.00, *p* < 0.0013) and residual variance of .9880 (0.01), *Z* = 66.96, *p* < 0.0001)]. The intraclass correlation suggested that 15% of Penetration variance was due to variation between treatment sites, indicating that multilevel analysis was appropriate.

#### H4: penetration over time, across conditions

Findings for H4 (penetration) were similar to H1 (service receipt). The main effect for Study Phase was significant, documenting an increase in Cascade Penetration over time [*F*(4,7973) = 11.77, *p* < 0.001, ES = 0.024; see Table 4]. Of the covariates, neither sex (male), urbanicity, nor level of charge was significant. However, Race (White vs. non-White) [*F*(1,8071) = 21.69, *p* < 0.0001], probation [*F*(1,7973) = 235.51, *p* < 0.0001], and positive urine [*F*(1,7973) = 52.07, *p* < 0.0001] were significant; with each having a positive relationship to Penetration. The least square means (see Table 5) show significant contrasts between Baseline and Early Experiment (*t* = − 4.76, *p* < 0.0001) and Baseline and Late Experiment (*t* = − 4.27, *p* < 0.0001). The contrast between Baseline and Maintenance was not significant (*t* = 1.12, *p* = 0.26). Overall, results supported H4 that there would be a significant increase in penetration for Early and Late Experiment compared to Baseline.

**Table 4:** The Type III Fixed Effects of Multilevel Analysis of Cascade Penetration for H4-H5

	Multilevel analysis with covariates for H4^**a**^		Multilevel analysis with covariates for H5^**b**^	
	*df*	*F-test (p)*	*df*	*F-test (p)*
Sitepairs	9,10	0.82 (.61)	9,9	0.75 (.66)
Core+Enhanced Condition (C) ^c^	NA	NA	1,7969	0.00 (.96)
Study Phase (SP)	4,7973	11.77 **(< .0001**)	4,7969	11.40 **(<.0001)**
C x SP	NA	NA	4,7969	2.88 **(.02)**
Male	1,7973	1.62 (0.20)	1,7969	1.73 (.19)
White	1,7973	21.69 **(< .0001)**	1,7969	21.74 **(< .0001)**
Probation	1,7973	235.51 **(< .0001)**	1,7969	234.99 **(< .0001)**
Positive Urine	1,7973	52.07 **(< .0001)**	1,7969	51.11 **(< .0001)**
Maximum Charge Level	1,7973	0.35 (.55)	1,7969	0.40 (.53)
% Urbanicity	1,7973	1.24 (.27)	1,7969	1.08 (.30)

*Note.* Probation = Whether or not being on probation. Positive Urine = Whether or not having a positive urine screen. Charges = Number of charges. % Urbanicity = Percentage of residents in urban areas. **Bold if** ***p*** **< .05**

^a^ site variance (0.16 (0.07), *Z* = 2.04, *p* = 0.02); residual variance = 0.91 (0.01) (*Z* = 63.14, *p* < 0.0001); model *χ*^*2*^(1) = 543.12, *p* < 0.0001)

^b^ site variance (0.18 (0.09), Z = 1.93, *p* = 0.03), residual variance =0.91 (.01), *Z* = 63.13, *p* < 0.0001. model *χ*^*2*^(1) = 407.83, *p* < 0.0001

^c^ Core condition = 0, Core + Enhanced Condition = 1

H4: SP Effect size = .024; H5: CxSP Effect size = .022

**Table 5:** Least Square Means (M), Standard Errors (SE), and Contrasts for Cascade Penetration with Covariates (H4)

***Contrast***	M	SE	***t***	***p***	Lower 95% CI	Upper 95%CI	Effect size ***d***
*Baseline Phase*	2.42	0.16	Reference				
*Pre-Randomization Phase* ^*a*^	2.52	0.17	NA	NA	–	–	
*Early Experiment Phase*	2.57	0.17	−4.76	**< 0.0001**	0.09	0.22	**0.16**
*Late Experiment Phase*	2.56	0.17	−4.27	**< 0.0001**	0.08	0.21	**0.14**
*Maintenance Phase*	2.38	0.11	1.12	0.26	−0.11	0.02	**0.04**

*Note*. ^a^ The contrast between Baseline Phase and Pre-Randomization Phase was omitted because it was not involved in hypothesis testing. 95% CI = 95% confidence intervals for the differences between least square means for the contrasts

#### H5: penetration over time, by condition

H5 extended the Penetration analyses to include Condition, Study Phase, and their interaction with Site pairs as the blocking factor, controlling for covariates. Study Phase [*F*(4,7969) = 11.40, *p* < 0.0001] and Study Phase x Condition were significant [*F*(4,7969) = 2.88, *p* = 0.02, ES = .022]. In addressing simple effects for the significant interaction, post-hoc tests revealed one significant change from Baseline to Late Experiment (*t* = − 1.97, *p <* 0.05) in the Core condition (see Table 6). However, for Core+Enhanced, there were significant increases from Baseline to Early Experiment (*t* = − 4.83, *p* < 0.0001) and from the Baseline to Late Experiment (*t* = − 4,07, *p* < 0.0001). No significant change occurred between Baseline and Maintenance (*t* = 1.08, *p* = 0.28). Overall, there was statistical support for H5. While there was some increase in Penetration in Core, there was greater Penetration over time in Core+Enhanced.

**Table 6:** Contrasts between three Study Phases on Cascade Penetration while Controlling for Covariates (H5)

Study Phase	CORE Condition						CORE + ENHANCED Condition					
*Contrast*	*M (SE)*^*a*^	*t*	*p*	*Lower 95% CI*	*Upper 95% CI*	***d***	*M (SE)*^*a*^	*t*	*p*	*Lower 95% CI*	*Upper 95% CI*	***d***
Baseline Phase	2.43 (0.23)						2.40 (0.19)					
Early Experiment Phase	2.52 (0.23)	−1.87	**0.06**	−0.17	0.01	**0.09**	2.62 (0.19)	−4.83	**< 0.0001**	−0.30	−0.13	**0.22**
Late Experiment Phase	2.53 (0.23)	−1.97	**0.05**	−0.18	0.01	**0.09**	2.59 (0.19)	−4.07	**< 0.0001**	−0.28	−0.10	**0.19**
Maintenance Phase (2.41, 0.23)	2.41 (0.23)	0.46	0.64	−0.06	0.13	**0.02**	2.35 (0.19)	1.08	0.28	−0.05	0.14	**0.05**

*Note*. ^a^ Least Square Means (M) and standard errors (SE) of cascade penetration at each study phase per intervention type. The contrast between Baseline Phase and Pre-Randomization Phase was omitted because it was not involved in hypothesis testing

## Discussion

Despite considerable variability across justice agencies in Cascade-related practices, this investigation detected an impact of the JJ-TRIALS intervention on youth treatment service outcomes, adjusting for youth, justice, and county characteristics. Findings document that Core intervention strategies (training teams on the BH Services Cascade, DDDM, and supporting goal selection) were effective at increasing service receipt over time relative to baseline (H1). When comparing cohorts of youth who entered during different study periods, Initiation rates increased 5.7% from Baseline to Early/Late Experiment. Engagement and Continuing Care rates increased significantly between Baseline and Early Experiment periods and extended to later periods. There was no difference between Core and Core+Enhanced conditions on receipt of treatment services over time (H2), neither study phase nor its interaction with condition was significant. For H3, youth initiated treatment 51.3% faster in Core+Enhanced sites than in Core sites (H3).

Findings also document that Cascade Penetration (last service stage achieved) increased from Baseline to Early and Late experiment in both conditions (H4), and the Core+Enhanced intervention (external facilitation of team progress toward goals using DDDM) was more effective in Penetration across study periods compared to Core (H5). These data suggest that efforts to improve services along the Cascade are more effective when teams work iteratively and collaboratively with external BH providers and have the guidance, support, and accountability that external facilitation provides. This type of outer/inner context collaboration is one critical bridging factor that can lead to more effective system-level implementation efforts [47]. It is worth noting, however, that improvement was modest even within Core+Enhanced sites; most youth in need did not receive treatment. Given higher costs associated with Core+Enhanced strategies, future research should examine its cost-effectiveness compared to Core.

This study extends prior research on SU Referral (under the purview of JJ) to services most often delivered by community partners. The pattern showing greater increases in referral rates over time in Core+Enhanced sites [39] was similar to findings for Penetration in this study but was not replicated for treatment service receipt. A number of distinctions between these two investigations may explain the differences. The Belenko et al. study included 30 sites with data through referral, whereas this study included 20 sites with data covering the full Cascade. The smaller sample included sites that prioritized documenting service utilization outside of JJ and could differ in terms of inter-organizational collaboration and capacity to implement change [4, 5, 48]. Moreover, treatment referral was at the discretion of the JJ agency and may have been particularly sensitive to the JJ-TRIALS intervention. The focus was primarily on JJ system change, with representation from BH on implementation teams to provide consultation and support. Expectations that BH agencies would modify their programs and practices (not specific to JJ referrals) were only implied. Future efforts should attempt to balance these partnerships or expand to focus on BH system change, with consultation from JJ. Additional implementation supports that could foster greater sustainment might include a longer intervention period (one year may be insufficient to develop effective cross-system coordination); financial and technical support for improving data infrastructure and quality; virtual tutorials or technical assistance in using data in real time (e.g., charting trends using MIS records); additional training and incentives for probation officers to engage with BH providers; and ongoing feedback for probation officers (based on records audits) regarding service receipt among youth on their caseloads. Beyond these implementation factors, later Cascade events are likely influenced by a complex set of determinants, including JJ sanctions, BH waitlists, and youth/family considerations (e.g., motivation, transportation barriers, family disorganization). Therefore, future studies should also consider other outcomes related to treatment Engagement such as parental support, motivation for change, adjudication and disposition decisions, etc.

Perhaps the predominant finding was the ubiquity of site variability across jurisdictions, target services, and implementation over time. Observing between-site differences is more rule than exception for multisite behavioral intervention trials [49], at least in JJ settings given the variability in legal codes and jurisprudence goals [42]. Although outcomes research characteristically places site variability in the background (via statistical control procedures) in order to emphasize generalizability of results, it can be equally illuminating to foreground site differences to better understand dynamic interactions of salient inner and outer context factors on targeted outcomes. Future studies should consider using individual-differences analytic approaches [50] and leverage qualitative data to investigate the myriad of causes of and impacts on site variability. For example, variations in how sites determined need for SU treatment (e.g., corroborating evidence from two or more sources versus relying on a single source) could affect referral decisions and Cascade retention. As described herein, the Cascade assumes that youth will ideally remain in services through continuing care; however, some referrals may be deemed inappropriate by treatment staff (e.g., when referral is based primarily on parent perception). Future studies should attempt to address the possibility of appropriate exit out of the Cascade through prospective data collection and/or qualitative inquiry.

Site variability highlights the need to further understand change processes, including strategies used by implementation teams as they planned and orchestrated system change [51]. For example, implementation teams in some Core+Enhanced sites spent months developing and refining tools to standardize and inform referral decisions, whereas some piloted changes before implementing practices agency-wide. Such activities might have produced qualitative improvements (e.g., more efficient and accurate identification) that take months or years to be reflected in service receipt patterns. While some Core sites applied DDDM, many tackled simpler modifications to JJ practices with immediate benefits such as modifying referral forms or offering JJ office space to contracted service providers. While contracting between outer and inner context service organizations is an EPIS bridging factor, the nature of contracts, including language, statements or work, and fiscal incentives, may affect service results [18, 47].

Sustainment is a critical concern in implementation research, and beginning with sustainment in mind – as a critical goal – cannot be understated [18]. While the expectation of continued gains during the Maintenance study period was not stated explicitly in hypotheses, EPIS-inspired study design and intervention components included an emphasis on sustainment [20]. In these data, means for Maintenance cohorts were low across Cascade events, likely because the study concluded without adequate time for youth entering sites to progress past screening/assessment. The degree to which practice changes were sustained after the study concluded is unknown. Future work should examine how gains attained in demonstration projects can be institutionalized in service systems [52, 53]. Attaining sustainment requires going beyond usual funding and accountability mechanisms (e.g., grants) to consider how practices and approaches fit within the complex and dynamic contexts of the broader system.

## Limitations

It is important to acknowledge potential limitations. First, it is not certain how much service receipt data were missing and for what reasons (e.g., not collected, not available) [13], nor the degree to which use of dichotomous measures limited power to detect significant effects. Records data include errors that may operate in multiple directions, and summarizing over multiple items helps to cancel out random error and focus on the underlying signal or “effect.” Summarizing across Cascade steps within a site (i.e., Cascade Penetration) results in a more stable estimate of the effects within (and therefore across) sites due to less variance, larger effects, and consequently more power than analyses focused on individual steps. To sufficiently identify and address existing barriers to Cascade penetration and determine the success of change efforts, JJ systems must invest in quality data captured throughout the Cascade, incorporating data from BH providers where feasible.

Second, the sample was not nationally representative, so findings may not generalize to other jurisdictions; however, youth and agency characteristics were sufficiently diverse and comparable to a nationally representative survey of JJ agencies [3]. Third, the subset of sites that collected data across the full Cascade may have better relationships with service providers compared to those who do not capture that data. They could also have leadership at the facility, county, and/or state level that prioritized and funded SU and/or other services. Fourth, the EPIS-inspired study design examined change over time by assigning cohorts of youth to study phases based on date of entry into the system. The lower means among the maintenance cohort could be attributed to the shorter time span available for youth to receive services. For example, receipt of services for youth in the Baseline cohort occurred over a 2-year period, compared to only 6 months (and sometimes less for youth entering late in the period) for youth in the Maintenance cohort. Given that on average youth were screened 16 days after JJ entry and 156 days elapsed between Screening and treatment Initiation, many youth in Maintenance (and some in Late Experiment) may have initiated after the study concluded. Finally, several measures that could potentially help explain these findings were not captured in the records (e.g., change in youth supervision status over time, positive random urine screenings, history of SU or mental health treatment).

## Conclusion

Results from this study demonstrate that change is possible in complex service systems involving JJ and BH agencies. Improvement in SU treatment initiation occurred across Core and Core+Enhanced conditions, the former without external facilitation. Furthermore, Core implementation activities appear beneficial even when instruction is not specific to an evidence-based practice and target goals are allowed to vary along the Cascade. Better Cascade penetration occurs when external facilitation is provided, but wide variation exists in the degree and nature of change across service systems.

Findings demonstrate the criticality of early EPIS phases from exploration through implementation, suggesting that Core strategies provided early on are effective at producing some improvement in treatment initiation rates. Implementation-focused activities in the Core+Enhanced condition (facilitation of implementation teams) are effective at incremental improvement in moving youth farther along the Cascade. Therefore, using a collaborative, multi-agency approach to system change, that utilizes a data-driven approach (with or without intensive facilitation), can be useful in improving SU treatment initiation rates, although substantial gaps remain in engaging youth in treatment after initiation. Focusing on improving SU identification and service receipt among justice-involved youth translates to benefits for public health and public safety.

## Data Availability

The current protocol is part of the Juvenile-Justice Translational Research on Interventions for Adolescents in the Legal System (JJ-TRIALS) Cooperative with a total of six research centers and one Coordinating Center. All data were de-identified (e.g., by systematically scrambling Study IDs within study sites) before master datafiles combined data across the six centers. At project close, data will be shared publicly, following NIDA guidance for appropriate public access and data sharing plans. Sharing of underlying primary data for publications will be made broadly available through an appropriate data repository.

## Abbreviations

BH: Behavioral Health
Cascade: Behavioral Health Services Cascade
EPIS: Exploration, Preparation, Implementation, and Sustainment
JJ: Juvenile Justice
JJ-TRIALS: Juvenile Justice-Translational Research for Adolescents in the Justice System
SU: Substance use
PDSA: Play-Do-Study-Act
DDDM: Data-Driven Decision Making

## References

[1] Doran N, Luczak SE, Bekman N, Koutsenok I, Brown SA (2012). Adolescent substance use and aggression: a review. Crim Justice Behav.

[2] Weber S, Lynch S (2021). Understanding the relations among adverse childhood experiences (ACE), substance use, and reoffending among detained youth. Child Abuse Negl.

[3] Scott CK, Dennis ML, Grella CE, Funk RR, Lurigio AJ. Juvenile justice systems of care: results of a national survey of community supervision agencies and behavioral health providers on services provision and cross-system interactions. Heal justice. 2019;7(1). 10.1186/s40352-019-0093-x. 10.1186/s40352-019-0093-x PMC6717998 31201642

[4] Welsh WN, Dembo R, Lehman WEK, Bartkowski JP, Hamilton L, Leukefeld CG (2021). Critical factors influencing Interorganizational relationships between juvenile probation and behavioral health agencies. Adm Policy Ment Heal Ment Heal Serv Res.

[5] Wasserman GA, McReynolds LS, Taxman FS, Belenko S, Elkington KS, Robertson AA (2021). The missing link (age): multilevel contributors to service uptake failure among youths on community justice supervision. Psychiatr Serv.

[6] Tolou-Shams M, Brown LK, Marshall BDL, Dauria E, Koinis-Mitchell D, Kemp K (2019). The behavioral health needs of first-time offending justice-involved youth: substance use, sexual risk, and mental health. J Child Adolesc Subst Abuse.

[7] Wasserman Larkin GA, Mcreynolds S, Schwalbe CS, Keating JM, Jones SA (2010). Psychiatric disorder, comorbidity, and suicidal behavior in juvenile justice youth. Crim Justice Behav.

[8] Bowser D, Henry BF, Wasserman GA, Knight D, Gardner S, Krupka K (2018). Comparison of the Overlap between Juvenile Justice Processing and Behavioral Health Screening, Assessment and Referral. J Appl Juv Justice Serv.

[9] Puzzanchera C, Adams B. National Report Series: juvenile arrests 2009. Office of Justice Programs. Washington, D.C.; 2011. Available from https://ojjdp.ojp.gov/sites/g/files/xyckuh176/files/pubs/236477.pdf.

[10] Hogue A, Bobek M, Levy S, Henderson CE, Fishman M, Becker SJ (2021). Conceptual framework for telehealth strategies to increase family involvement in treatment and recovery for youth opioid use disorder. J Marital Fam Ther.

[11] Chang DC, Klimas J, Wood E, Fairbairn N (2018). Medication-assisted treatment for youth with opioid use disorder: current dilemmas and remaining questions. Am J Drug Alcohol Abuse..

[12] Fishman M, Wenzel K, Scodes J, Pavlicova M, Lee JD, Rotrosen J (2020). Young adults have worse outcomes than older adults: secondary analysis of a medication trial for opioid use disorder. J Adolesc Health.

[13] Dennis ML, Smith CN, Belenko S, Mcreynolds L, Dembo R, Robertson A (2019). Operationalizing a behavioral health services Cascade of care model: lessons learned from a 33-site implementation in juvenile justice community supervision 1. Fed Probat.

[14] Belenko S, Knight D, Wasserman GA, Dennis ML, Wiley T, Taxman FS (2017). The juvenile justice behavioral health services Cascade: a new framework for measuring unmet substance use treatment services needs among adolescent offenders. J Subst Abus Treat.

[15] MacCarthy S, Hoffmann M, Ferguson L, Nunn A, Irvin R, Bangsberg D (2015). The HIV care cascade: models, measures and moving forward. J Int AIDS Soc.

[16] Williams AR, Nunes EV, Bisaga A, Levin FR, Olfson M (2019). Development of a Cascade of care for responding to the opioid epidemic. Am J Drug Alcohol Abuse.

[17] Aarons GA, Hurlburt M, Horwitz SMC (2011). Advancing a conceptual model of evidence-based practice implementation in public service sectors. Admin Pol Ment Health.

[18] Moullin JC, Dickson KS, Stadnick NA, Rabin B, Aarons GA (2019). Systematic review of the exploration, preparation, implementation, sustainment (EPIS) framework. Implement Sci.

[19] Knight DK, Belenko S, Wiley T, Robertson AA, Arrigona N, Dennis M, et al. Juvenile justice-translational research on interventions for Adolescents in the legal system (JJ-TRIALS): a cluster randomized trial targeting system-wide improvement in substance use services. Implement Sci. 2016;11(1). 10.1186/s13012-016-0423-5. 10.1186/s13012-016-0423-5 PMC4850663 27130175

[20] Becan JE, Bartkowski JP, Knight DK, Wiley TRA, DiClemente R, Ducharme L (2018). A model for rigorously applying the exploration, preparation, implementation, sustainment (EPIS) framework in the design and measurement of a large scale collaborative multi-site study. Heal Justice.

[21] Powell BJ, Waltz TJ, Chinman MJ, Damschroder LJ, Smith JL, Matthieu MM (2015). A refined compilation of implementation strategies: results from the expert recommendations for implementing change (ERIC) project. Implement Sci.

[22] Welsh WN, Knudsen HK, Knight K, Ducharme L, Pankow J, Urbine T (2016). Effects of an organizational linkage intervention on inter-organizational service coordination between probation/parole agencies and community treatment providers. Admin Pol Ment Health.

[23] Welsh WN, Prendergast M, Knight K, Knudsen H, Monico L, Gray J (2016). Correlates of Interorganizational service coordination in community corrections. Crim Justice Behav.

[24] Aarons GA, Palinkas LA (2007). Implementation of evidence-based practice in child welfare: service provider perspectives. Admin Pol Ment Health.

[25] Lawrence R (1995). Controlling school crime: an examination of Interorganizational relations of school and juvenile justice professionals. Juv Fam Court J.

[26] Smith BD, Mogro-Wilson C (2007). Multi-level influences on the practice of inter-agency collaboration in child welfare and substance abuse treatment. Child Youth Serv Rev.

[27] Howell JC, Kelly MR, Palmer J, Mangum RL. Integrating child welfare, juvenile justice, and other agencies in a continuum of services on JSTOR. Child Welfare. 2004:143–56 Available from: https://www.jstor.org/stable/45400318?seq=1#metadata_info_tab_contents. [cited 2022 Feb 28]. 15068216

[28] Belenko S, Visher C, Copenhaver M, Hiller M, Melnick G, O’connell D (2013). A cluster randomized trial of utilizing a local change team approach to improve the delivery of HIV services in correctional settings: study protocol. Heal Justice.

[29] Hoffman KA, Green CA, Ford JH, Wisdom JP, Gustafson DH, McCarty D (2012). Improving quality of care in substance abuse treatment using five key process improvement principles. J Behav Health Serv Res..

[30] Kirchner JAE, Parker LE, Bonner LM, Fickel JJ, Yano EM, Ritchie MJ (2012). Roles of managers, frontline staff and local champions, in implementing quality improvement: stakeholders’ perspectives. J Eval Clin Pract.

[31] Brynjolfsson E, McElheran K (2016). The rapid adoption of data-driven decision-making. Am Econ Rev.

[32] Mandinach EB (2012). A perfect time for data use: using data-driven decision making to inform practice. Educ Psychol.

[33] McKay C (2019). Predicting risk in criminal procedure: actuarial tools, algorithms, AI and judicial decision-making. Curr Issues Crim Justice.

[34] Witjas-Paalberends ER, van Laarhoven LPM, van de Burgwal LHM, Feilzer J, de Swart J, Claassen E (2018). Challenges and best practices for big data-driven healthcare innovations conducted by profit–non-profit partnerships–a quantitative prioritization. Int. J Healthc Manag.

[35] Taylor MJ, McNicholas C, Nicolay C, Darzi A, Bell D, Reed JE (2014). Systematic review of the application of the plan-do-study-act method to improve quality in healthcare. BMJ Qual Saf.

[36] Fixsen DL, Blase KA, Naoom SF, Wallace F (2009). Core Implementation Components. Res Soc Work Pract.

[37] Powell BJ, Warren G, Mcmillen JC, Proctor EK, Carpenter CR, Griffey RT (2011). A compilation of strategies for implementing clinical innovations in health and mental health. Med Care Res Rev.

[38] Marsh JA, Mccombs JS, Martorell F (2009). How instructional coaches support data-driven decision making policy implementation and effects in Florida middle schools. Educ Policy.

[39] Belenko S, Knight D, Dembo R, Wasserman G, Robertson A, Elkington K, et al. Effects of a bundled implementation intervention to improve assessment of treatment need and referral to substance use services among justice-involved youth: Findings from a multisite cluster randomized implementation trial. J Subst Abus Treat. 2017;82:107–12. https://pubmed.ncbi.nlm.nih.gov/29021108/. 10.1016/j.jsat.2022.108829 PMC9357202 35751945

[40] Fisher JH, Becan JE, Harris PW, Nager A, Baird-Thomas C, Hogue A, et al. Using goal achievement training in juvenile justice settings to improve substance use services for youth on community supervision. Heal justice. 2018;6(1). 10.1186/s40352-018-0067-4. 10.1186/s40352-018-0067-4 PMC5928026 29713840

[41] Becan JE, Fisher JH, Johnson ID, Bartkowski JP, Seaver R, Gardner SK (2020). Improving substance use Services for Juvenile Justice-Involved Youth: complexity of process improvement plans in a large scale multi-site study. Admin Pol Ment Health.

[42] Sickmund M, Puzzanchera C (2014). National Report Series: Juvenile Offenders and Victims 2014 National Report.

[43] Stokes M, Davis C, Koch G (2012). Categorical data analysis using SAS, third edition.

[44] Miller P (2020). Solved: Re: Effect size in GLIMMIX - SAS Support Communities.

[45] Lorah J (2018). Effect size measures for multilevel models: definition, interpretation, and TIMSS example. Large-Scale Assess Educ.

[46] Selya AS, Rose JS, Dierker LC, Hedeker D, Mermelstein RJ (2012). A practical guide to calculating Cohen’s f 2, a measure of local effect size, from PROC MIXED. Front Psychol.

[47] Lengnick-Hall R, Willging C, Hurlburt M, Fenwick K, Aarons GA, Aarons GA (2020). Contracting as a bridging factor linking outer and inner contexts during EBP implementation and sustainment: a prospective study across multiple U.S. public sector service systems. Implement Sci.

[48] Knight DK, Joe GW, Morse DT, Smith C, Knudsen H, Johnson I (2019). Organizational context and individual adaptability in promoting perceived importance and use of best practices for substance use. J Behav Health Serv Res.

[49] Ball SA, Martino S, Nich C, Frankforter TL, Van Horn D, Crits-Christoph P (2007). Site matters: multisite randomized trial of motivational enhancement therapy in community drug abuse clinics. J Consult Clin Psychol.

[50] Howard MC, Hoffman ME. Variable-Centered, Person-centered, and person-specific approaches: where theory meets the method Organ Res Methods 2018;21(4):846–876. 10.1177/1094428117744021.

[51] Lewis CC, Boyd MR, Walsh-Bailey C, Lyon AR, Beidas R, Mittman B (2020). A systematic review of empirical studies examining mechanisms of implementation in health. Implement Sci.

[52] Chaffin M, Hecht D, Aarons G, Fettes D, Hurlburt M, Ledesma K (2016). EBT Fidelity trajectories across training cohorts using the interagency collaborative team strategy. Admin Pol Ment Health.

[53] Hurlburt M, Aarons GA, Fettes D, Willging C, Gunderson L, Chaffin MJ (2014). Interagency collaborative team model for capacity building to scale-up evidence-based practice. Child Youth Serv Rev.

